# Perfluoroalkyl acid and bisphenol-A exposure via food sources in four First Nation communities in Quebec, Canada

**DOI:** 10.1017/S1368980022000581

**Published:** 2022-03-11

**Authors:** Claudelle Dubeau, Amira Aker, Élyse Caron-Beaudoin, Pierre Ayotte, Caty Blanchette, Nancy Gros-Louis McHugh, Mélanie Lemire

**Affiliations:** 1 Département de Médecine Sociale et Préventive, Institut de Biologie Intégrative et des Systèms, Université Laval, Québec, QC, Canada; 2 Axe Santé Des Populations et Pratiques Optimales en Santé, Centre de Recherche du CHU de Québec, Université Laval, 1050 Ch Ste-Foy, Québec, QC G1S 4L8, Canada; 3 Department of Health and Society, University of Toronto Scarborough, Toronto, ON, Canada; 4 Centre for Clinical Epidemiology and Evaluation, University of British Columbia, Vancouver Coastal Health Research Institute, Research Pavilion, Vancouver, BC, Canada; 5 Institut National de Santé Publique du Québec, Quebec, Canada; 6 Commission de Santé et de Services Sociaux Des Premières Nations Québec Labrador, Wendake, QC, Canada

**Keywords:** Perfluoroalkyl substances, Bisphenol A, Processed food, Food packaging, Traditional foods, Indigenous youth, First Nations

## Abstract

**Objective:**

To document perfluoroalkyl acids (PFAA) and bisphenol-A (BPA) exposure in four First Nation communities in northern Quebec compared with the Canadian Health Measures Survey (CHMS Cycle 5 2016–2017) and examine the associations between dietary consumption and chemical exposure.

**Design:**

We used cross-sectional data from the JES-YEH! project conducted in collaboration with four First Nation communities in 2015. A FFQ collected information on diet, and PFAA and BPA were measured in biological samples. We used generalised linear models to test the associations between food intake and chemical biomarkers.

**Setting:**

Northern Quebec.

**Participants:**

Youth aged 3–19 years (*n* 198).

**Results:**

Mean perfluorononanoic acid (PFNA) levels were significantly higher in JES-YEH! than CHMS, and BPA levels were higher among those aged 12–19 years compared with CHMS. Dairy products were associated with PFNA among Anishinabe and Innu participants (geometric mean ratio 95 % CI: 1·53 (95 % CI 1·03, 2·29) and 1·52 (95 % CI 1·05, 2·20), respectively). PFNA was also associated with ultra-processed foods (1·57 (95 % CI 1·07, 2·31)) among Anishinabe, and with wild fish and berries (1·44 (95 % CI 1·07, 1·94); 1·75 (95 % CI 1·30, 2·36)) among Innu. BPA was associated with cheese (1·72 (95 % CI 1·19, 2·50)) and milk (1·53 (95 % CI 1·02, 2·29)) among Anishinabe, and with desserts (1·71 (95 % CI 1·07, 2·74)), processed meats (1·55 (95 % CI 1·00, 2·38)), wild fish (1·64 (95 % CI 1·07, 2·49)) and wild berries (2·06 (95 % CI 1·37, 3·10)) among Innu.

**Conclusions:**

These results highlight the importance of better documenting food-processing and packaging methods, particularly for dairy products, and their contribution to endocrine disruptors exposures as well as to promote minimally processed and unpackaged foods to provide healthier food environments for youth in Indigenous communities and beyond.

Although traditional foods play a central role in Indigenous Peoples’ nutrition and culture, their consumption is increasingly being replaced by processed foods, particularly among younger generations^(1,2)^. These commercial foods are often lower in nutritional quality and higher in added sugar, salt and saturated fats^(3)^. This dietary transition, from a monotonous diet consisting mainly of minimally processed foods to a diverse diet dominated by highly processed foods, is believed to be the main cause of the increase in societal chronic diseases such as type 2 diabetes and obesity^(4)^. Indeed, several recent studies show a growing prevalence of obesity and type 2 diabetes among Indigenous youth in Quebec and elsewhere in Canada^(2,5)^. Furthermore, processed foods may also contain chemical contaminants from food processing and packaging such as perfluoroalkyl acids (PFAA) and bisphenol-A (BPA)^(6,7)^.

PFAA are a group of highly persistent chemicals whose production and use as water and oil repellents since the 1950s have led to the contamination of air, water and wildlife, and subsequent exposure in humans worldwide^(8,9)^. Due to the ubiquitous nature of PFAA, ambient exposures are prevalent and the majority of the population has some detectable PFAA concentrations. However, the main PFAA exposure sources include drinking water, food, food packaging, furniture, clothing, house dust and aerosols^(10)^. Paper and water/grease-resistant packaging, for example, have the potential to directly contaminate food with certain PFAA^(11,12)^. Microwave popcorn, butter, margarine, fast food, processed meat, dairy products and cookie consumption have all been associated with elevated levels of plasma PFAA^(7,8)^. To date, eight studies, including a few conducted in children, found a positive association between dairy intake and PFAA, three of which identified associations with exposure to perfluorononanoic acid (PFNA). While some older PFAA have seen their worldwide production and use restricted over the years, other more recent PFAA with longer carbon chains and their precursors are not fully regulated in Canada. Perfluorooctanesulfonic acid (PFOS; C8) was banned under the Stockholm Convention in 2009; perfluorooctanoic acid (PFOA; C8) was recently included in the Convention (May 2019); and, perfluorohexane sulphonic acid (PFHxS; C6) is still under review for inclusion^(13)^. In 2016, the Government of Canada amended Canada’s Prohibition of Certain Toxic Substances Regulations to add several PFAA, including those with longer chains such as PFNA (C9)^(14)^. Some PFAA, including PFOA and PFNA, are also degradation products of other neutral per- and polyfluoroalkyl substances such as fluorotelomer alcohols, which are still used as intermediates in many consumer and industrial products (e.g. paints, electronics, food paper packaging, etc.)^(15,16)^. These substances are called precursors, and are also regulated in Canada. Despite these regulations, it is possible that imported goods contain prohibited substances. Long-chain PFAA, their salts and their precursors were also nominated by Canada in 2021 and recently passed the Annex D requirements and moved to the next stage for inclusion in the list of chemicals under the Stockholm Convention^(13,17)^.

BPA is a synthetic non-persistent compound used in the production of polycarbonate plastic (reusable plastic containers, reusable water bottles, cups, etc.) and epoxy resins^(18–20)^. BPA was traditionally used in many food packaging materials, such as can linings, Mason jar lids, polycarbonate plastic containers and case receipts^(19,21–23)^; however, many manufacturers have replaced these materials for BPA-free plastics or resins. BPA has been measured in dairy products, pastries, processed meats and several canned foods, including legumes, soups, evaporated milk and baby food^(6,22,24,25)^. In 2012, a review of dietary and non-dietary exposures to BPA concluded that dietary sources contributed to over 99 % of overall BPA exposure in children aged 18 months to 5 years in the USA^(26)^. In 2010, Canada banned the manufacture, import, advertisement or sale of polycarbonate baby bottles containing BPA based on neurodevelopmental and behavioural health endpoints^(27)^.

BPA and PFAA are confirmed and/or suspected endocrine disruptors and are associated with several adverse paediatric and developmental outcomes^(28–31)^. More specifically, PFAA exposure in children are linked to thyroid hormone imbalances^(32–34)^, including in the First Nations Youth, Environment and Health Pilot Study (JES!-YEH!)^(34)^. BPA is thought to impact oestrogen function and is a suspected obesogen^(35–37)^.

JES!-YEH! is a biomonitoring initiative of environmental contaminants, nutritional status and other health determinants in Indigenous children and youth from four First Nation communities in Quebec, Canada. A significantly higher exposure to PFNA was reported among Anishinabe participants involved in JES!-YEH! compared with youth of the same age groups in the Canadian Health Measures Survey (CHMS) Cycle 2 (2009–2011), the only other Canadian data available for these age groups at that time^(34)^. To our knowledge, no studies have documented exposure to BPA among Indigenous youth in Canada.

The objective of the present study was to describe PFAA and BPA exposure among JES!-YEH! participants in comparison to CHMS data in youth, to document JES!-YEH! participants’ intake of different foods and to examine the associations between foods and chemical exposures. Traditional foods were also included as this was a concern expressed by our community partners.

## Methods

### Study population

**Fig. 1:**
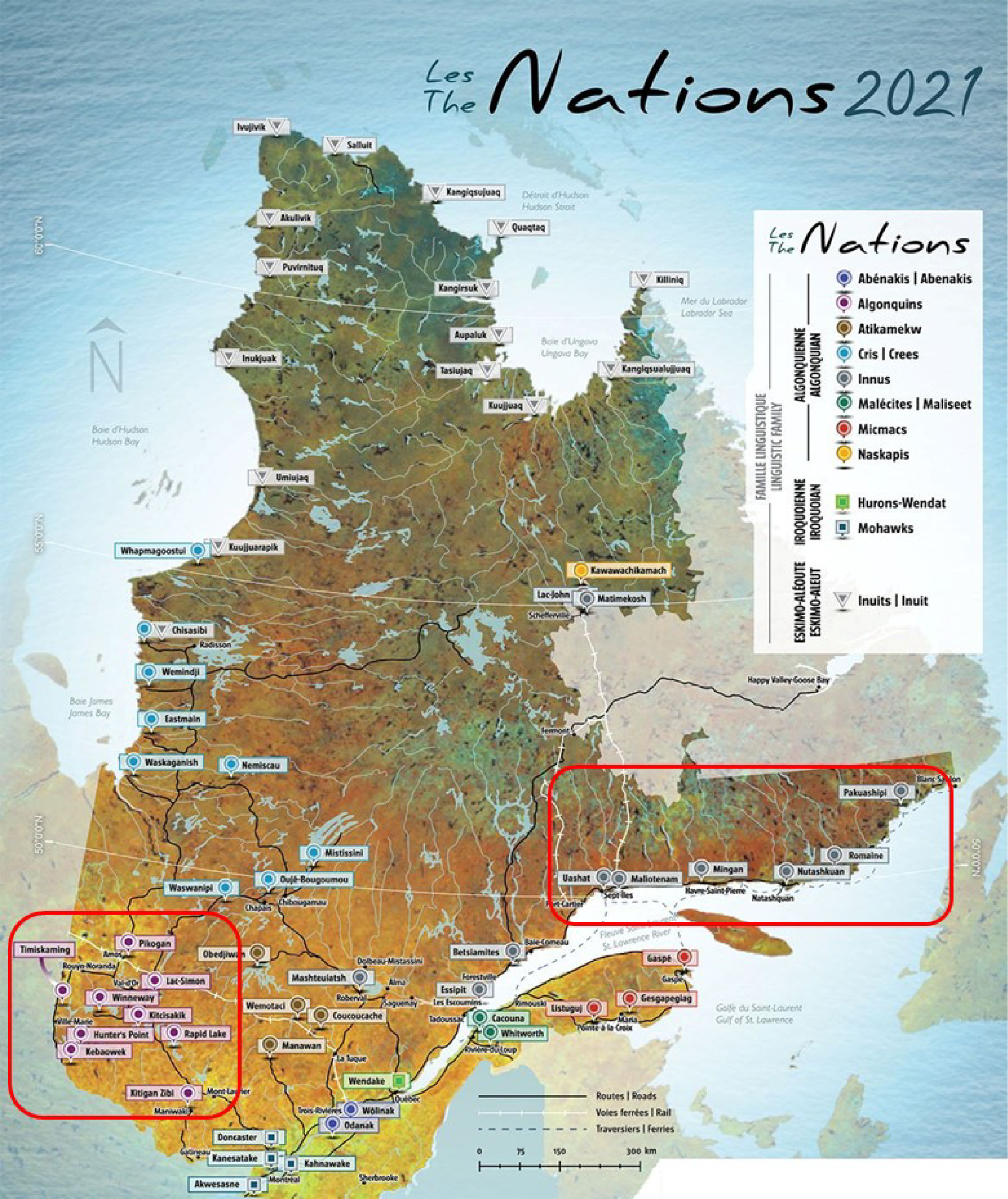
Map of the province of Quebec, Canada. The Abitibi-Temiscamingue (left) and Minganie and Lower North Shore (right) regions where the study took place are indicated by red rectangles. Algonquin nations in the figure are identified as Anishinabe in the manuscript according to participating communities’ preference

The JES!-YEH! project was realised in 2015 in collaboration with four First Nation communities in the province of Quebec (Fig. 1). This cross-sectional pilot study was conducted prior to the Food, Environment, Health and Nutrition of Children and Youth (FEHNCY) project, a pan-Canadian First Nations’ children and adolescent study initiated in 2019. A total of 198 children and adolescents aged 3–19 years were recruited from two Anishinabe communities in the Abitibi-Temiscamingue region (May and June 2015), and two Innu communities of Minganie and Lower North Shore regions (September and October 2015). Field research periods were suggested by community partners to minimise interference with hunting and fishing activities and important local events. Further details have been described elsewhere^(34)^. In short, the four community partners provided a list of 279 potential participants according to the population distribution characteristics (age categories: 3–5, 6–11 and 12–19 years for each sex) in each of the four communities based on the 2014 Statistics Canada Census^(38)^, from which 177 participants were randomly selected. An additional twenty-one participants were recruited on a voluntary basis and in accordance with the recruitment targets by age and sex to reach our recruitment target, totalling 198 participants. Children aged 3–17 years provided verbal consent and signed a consent form alongside their parent or guardian, who were also present during data collection. Young adults aged 18–19 years signed a consent form on their own behalf.

### Data collection

Two nurses collected anthropometric measurements, blood samples by venipuncture and spot urine samples. Participants and/or their parents or legal guardians were invited to answer an interview-administered questionnaire which included a traditional and market FFQ. The entire session lasted approximately 1·5 h. To facilitate recruitment, participants were not required to fast prior to data collection. Consent forms and questionnaires were completed in French or English according to the parent, guardian or participant’s preference. Local staff provided simultaneous translation in Anishinabe or Innu language as needed. Participants were provided a $50 food voucher as compensation.

Blood specimens were collected in a 10-mL serum determination tube (Red cap silicone-coated interior tube with a clot activator). Blood tubes were kept at room temperature for 0·5–1 h before being centrifuged at 6000 rpm for 15 min at room temperature. Serum samples were then aliquoted into 2 ml Sarstedt vials labelled for serum PFAA analyses and readily stored at –20°C. Urine spot samples were collected in a 60-ml polypropylene container and aliquoted into 3·5 ml samples in Sarstedt tubes for BPA and creatinine analyses, and stored at –20°C. All samples were transported frozen to the different laboratory facilities for further analyses.

### Chemical analyses of PFAA and BPA

Serum PFAA, urinary BPA and creatinine were analysed at the Centre de toxicologie du Québec of the Institut national de santé publique du Québec (INSPQ). Four PFAA (PFOS, PFOA, PFHxS and PFNA) were analysed in serum samples by Ultra Performance Liquid Chromatography (UPLC Waters Acquity) with a tandem mass spectrometer (MS/MS Waters Xevo TQ-S) in the multiple reaction monitoring mode with an electrospray ion source in the negative mode. Information on the methods used for the analysis of PFAA is described elsewhere^(34)^. The limits of detection (LOD) for PFOS, PFOA, PFHxS and PFNA were 0·03, 0·2, 0·04 and 0·07 μg/l, respectively. The intra-day precision varied between 3·3 and 8·1 % and the inter-day precision varied between 4·2 and 13 %, depending on the analytes. The calibration curve was made in bovine serum and was linear with a weighting of 1/x between 0·15 and 50 μg/l for PFNA, PFOA, PFHxS and between 0·6 and 200 μg/l for PFOS. The internal reference materials used to control the quality of the analyses were the certified reference material SRM-1958 from the National Institute of Standards and Technology (NIST) and some in-house quality controls for PFAA.

BPA was measured in urine at the Centre de toxicologie du Québec by GC – tandem MS (GC-MS/MS, INSPQ Method E-454) with an LOD of 0·2 µg/l. BPA concentrations are reported as a function of urine volume (µg BPA/l of urine) and adjusted for urinary creatinine (µg BPA/g of creatinine). Urinary creatinine concentrations were also analysed in the Centre de toxicologie du Québec and its detection limit was 0·0316 g/l. The creatinine in urine was measured with a DRI^TM^ Creatinine-Detect kit from Microgenics Corporation by spectrophotometry at a wavelength of 510 nm with an Analyzer Indiko Plus (ThermoFisher Scientific). Values below the LOD were divided by 2. Only one participant had a PFOA value<LOD and nine participants had a value <LOD for BPA.

### Food intake

The FFQ assessed traditional food consumption frequency by season over the last year, and market food and beverage consumption frequency over the past 3 months (spring for Anishinabe and summer/fall for Innu communities). Questions were developed based on previous First Nations and Inuit studies^(39,40)^, and piloted with community partners and volunteers from Anishinabe and Innu nations. Posters with images and names in English, French, Anishinabe or Innu languages of all traditional foods were used to provide a visual support. The present study only used data on traditional food consumption in the spring for Anishinabe communities and summer/fall for Innu communities to account for the time of biological sample collection. Food intake in grams per day (g/d) was calculated based on the age and sex of the participant^(2)^. Traditional and market food consumption were further grouped into categories of suspected PFAA or BPA exposure (see online Supplemental Tables S1–S5).

### Statistical analyses

Descriptive analyses and Chi-squared tests were used to describe the study population and test for significant differences by nation, age, sex and BMI. Chemical exposure variables were log-transformed given the skewedness of their distributions. Serum PFAA levels (µg/l) and urine BPA levels (µg/g creatinine) were compared by nation and age, and to those reported in the CHMS (Cycle 5, 2016–2017)^(41)^. Differences between JES!-YEH! and CHMS were considered statistically different if the 95 % CI of the geometric means (GM) did not overlap. The proportion of JES!-YEH! participants with serum levels of PFAA and urinary levels of BPA above the CHMS 95^th^ percentile (Cycle 5, 2016–2017) were also calculated. Chi-squared tests were used to test whether the proportion of JES!-YEH! participants above the CHMS 95^th^ percentiles was the same as CHMS (5 %). For food intake, medians (10^th^–95^th^ percentiles) were reported, and Kruskall–Walis tests were used to assess differences between age categories and Wilcoxon tests were used to assess differences between nations.

For the remaining analyses, each food category or item was dichotomised into low and high food consumption groups according to the median food intake in g/d: low (≤ median) and high (> median). If the median for a food intake category or item was equal to zero (food intake too low), the sample was dropped from the model. The associations between the dichotomous food category/item variables and chemical levels were investigated for sixteen food categories (see online Supplemental Tables S4 and S5) and five food items (milk, cheese, yoghurt, microwave popcorn and eggs) independently, based on a literature review of the food categories and items reported to contain PFAA or BPA (see online Supplemental Table S1, S2 and S3). All models were adjusted for age and sex^(7,42,43)^. To account for potential curvilinear effects of age on PFAA exposures, age-squared was also included in models^(34)^. Urinary creatinine was included in the models with BPA (µg/l) to adjust for urine level. All models were further stratified by nation.

From these models, the adjusted GM of each chemical by food category/item (low and high) were calculated by exponentiating the least-square means. The ratios of the two adjusted GM (chemical GM in the high food intake group/chemical GM in the low food intake group) and associated 95 % CI were also calculated. If the GM ratio was above one, the contaminant exposure was considered higher among those that reported consuming a high intake of that food category or item. For increased brevity, only food items associated with at least one chemical are presented. All analyses were performed using SAS 9.4 software.

A sensitivity analysis was performed by removing Anishinabe participants aged 6–11 years in models examining associations of food category/items with serum PFNA levels. This was due to the markedly high levels of serum PFNA levels in this age group compared with the levels in other Anishinabe age groups, Innu participants and CHMS Cycle 5.

## Results

Of the 198 participants in the JES!-YEH! study, 185 participants had complete data for food categories/items and the chemicals of interest (107 Anishinabe and 78 Innu participants) (Table 1). The median age was 10 years and 52·4 % of participants were boys. Almost two-thirds of the participants were considered overweight or obese. Innu participants were more likely to be obese (62·8 % *v*. 20·1 %), but there were no differences in age and sex distributions across the nations.

**Table 1:** Characteristics of the JES!-YEH! Participants from four Anishinabe and Innu communities in Quebec, Canada (*n* 185)

Characteristic	Total (*n* 185)		Anishinabe participants (*n* 107)		Innu participants (*n* 78)	
	*n*	%	*n*	%	*n*	%
Age						
3–5 years	36	19·5	23	21·5	13	16·7
6–11 years	73	39·5	45	42·1	28	35·9
12–19 years	76	41·1	39	36·4	37	47·4
Sex						
Female	88	47·6	53	49·5	35	44·9
Male	97	52·4	54	50·5	43	55·1
BMI category (kg/m^2^)*						
Underweight (<18·5)	2	1·1	2	1·2	0	
Normal (18·5–22·9)	60	32·4	46	43·0	14	17·9
Overweight (23–25)	50	27·0	37	34·6	13	16·7
Obese (>25)	71	38·4	22	20·1	49	62·8

* Chi-squared test *P*-value < 0·05.

**Fig. 2:**
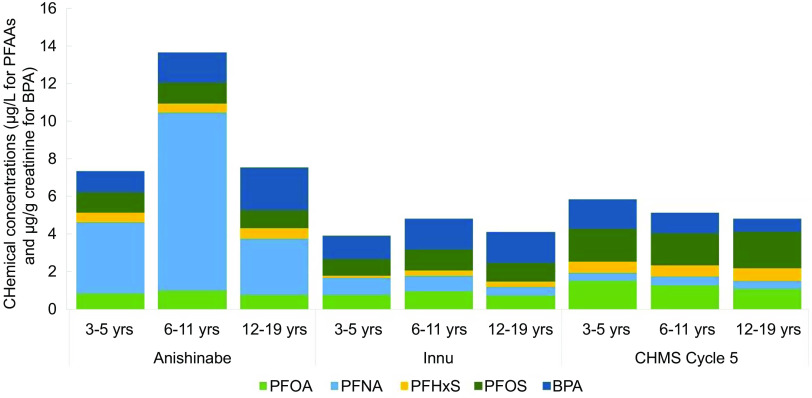
Chemical concentrations by age and nation groups in JES-YEH! compared with the CHMS Cycle 5

Serum PFNA and PFHxS were significantly higher among all Anishinabe participants compared with Innu participants in JES!-YEH! (PFNA GM: 5·12 µg/l (95 % CI: 4·28, 6·11) *v*. 0·64 µg/l (95 % CI 0·54, 0·76); PFHxS GM: 0·53 µg/l (95 % CI 0·48, 0·59) *v*. 0·25 µg/l (95 % CI 0·22, 0·28)) (Table 2 and Table S6, Fig. 2). Serum PFNA levels in Anishinabe participants were also 7–21 times higher than serum PFNA levels in CHMS Cycle 5. Alternatively, serum PFOS and PFOA levels were higher in CHMS *v*. participants in either nation. Serum PFHxS and urinary BPA levels in CHMS were on par with Anishinabe levels, except for higher urinary BPA levels among those aged 12–19 years in Anishinabe and Innu participants compared with CHMS. None of the serum PFAA or BPA levels differed by sex (data not shown).

**Table 2:** Serum levels of perfluorononanoic acid (PFNA), perfluorooctane sulfonate (PFOS), perfluorooctanoic acid (PFOA) and perfluorohexane sulfonate (PFHxS) (µg/l) and urine levels of bisphenol A (BPA) (μg/g creatinine) in participants from JES!-YEH! (2015), by nation and age groups, compared with the general Canadian population (CHMS cycle 5, 2016–2017)

	JES!-YEH!																					
	Anishinabe								Innu								CHMS cycle 5					
	GM	95 % CI	GM	95 % CI	GM	95 % CI	GM	95 % CI	GM	95 % CI	GM	95 % CI	GM	95 % CI	GM	95 % CI	GM	95 % CI	GM	95 % CI	GM	95 % CI
	Total (*n* 107)		3–5 years (*n* 23)		6–11 years (*n* 45)		12–19 years (*n* 39)		Total (*n* 78)		3–5 years (*n* 13)		6–11 years (*n* 28)		12–19 years (*n* 37)		3–5 years*		6–11 years†		12–19 years‡	
PFNA	5·12	4·28, 6·11	3·80^a^	2·65, 5·45	9·44^b^	8·12, 10·97	3·01^a^	2·20, 4·11	0·64	0·54, 0·76	0·89^a^	0·44, 1·80	0·86^a^	0·67, 1·11	0·46^b^	0·39, 0·54	0·45^A^	0·40, 0·51	0·45^AI^	0·37, 0·53	0·41^A^	0·33, 0·51
PFOS	1·03	0·94, 1·12	1·05	0·82, 1·33	1·09	0·97, 1·22	0·95	0·81, 1·12	1·01	0·91, 1·11	0·86^a^	0·72, 1·04	1·12^b^	0·99, 1·27	0·98^ab^	0·81, 1·18	1·70^AI^	1·50, 2·10	1·70^AI^	1·50, 2·00	1·91^AI^	1·70, 2·00
PFOA	0·87	0·81, 0·94	0·85^ab^	0·69, 1·06	1·02^a^	0·93, 1·11	0·75^b^	0·66, 0·85	0·81	0·76, 0·86	0·78^a^	0·68, 0·90	0·95^b^	0·84, 1·07	0·73^a^	0·68, 0·79	1·50^AI^	1·30, 1·60	1·30^AI^	1·20, 1·40	1·10^AI^	1·95, 1·20
PFHxS	0·53	0·48, 0·59	0·51	0·38, 0·68	0·50	0·44, 0·58	0·57	0·48, 0·68	0·25	0·22, 0·28	0·14^a^	0·12, 0·18	0·26^b^	0·22, 0·31	0·29^b^	0·24, 0·34	0·61^I^	0·46, 0·81	0·59^I^	0·45, 0·77	0·69^I^	0·59, 0·80
BPA	1·70	1·35, 2·15	1·13^a^	0·72, 1·77	1·61^a^	1·16, 2·23	2·29^b^	1·46, 3·59	1·58	1·25, 1·99	1·26	0·82, 1·93	1·64	1·09, 2·44	1·66	1·14, 2·42	1·60	1·40, 1·90	1·10	0·99, 1·30	0·74^AI^	0·58, 0·94

* PFNA (*n* 453), PFOS (*n* 491), PFOA (*n* 491), PFHxS (*n* 491), BPA (*n* 547).

† PFNA (*n* 492), PFOS (*n* 520), PFOA (*n* 520), PFHxS (*n* 520), BPA (*n* 516).

‡ PFNA (*n* 494), PFOS (*n* 527), PFOA (*n* 527), PFHxS (*n* 527), BPA (*n* 524).

Geometric means (95 % CI).

Bolded estimates represent a significant difference in contaminant levels between participants from Anishinabe and Innu nations (*P* < 0·05).

Lowercase a and b represent a significant difference in contaminant levels between participants across age groups in each nation (*P* < 0·05); i.e. groups with the same letter are not significantly different and groups with different letters are significantly different.

Uppercase A and I represent a significant difference in contaminant levels between participants in CHMS Cycle 5 and each nation (confidence intervals do not overlap) such that A represents Anishinabe and I represents Innu.

Across age groups in each nation, serum PFNA levels were most strikingly higher among participants aged 6–11 years in Anishinabe compared with other age categories (9·44 µg/l (95 % CI 8·12, 10·97) *v*. 3·80 µg/l (95 % CI 2·65, 5·45) and 3·01 µg/l (95 % CI 2·20, 4·11) in participants aged 3–5 and 12–19 years) (Table 2). Conversely, serum PFNA levels were similar in those aged 3–5 and 6–11 years among Innu (0·89 µg/l (95 % CI 0·44, 1·80) and 0·86 µg/l (95 % CI 0·67, 1·11)), and lowest in those aged 12–19 years (0·46 µg/l (95 % CI 0·39, 0·54)). Among Anishinabe, serum PFOA levels were also slightly higher in those aged 6–11 years compared with those aged 12–19 years and urinary BPA levels increased with age. Among Innu, serum PFOS and PFOA levels were higher in those aged 6–11 years *v*. 3–5 years, and PFHxS levels increased with age. The proportion of JES!-YEH! participants with serum levels of PFNA and urinary levels of BPA over the CHMS Cycle 5 95^th^ percentile ranged between 19 and 78 %, which was significantly higher than the expected 5 % (see online supplemental Table S7).

Dairy products were consumed in larger quantities among all participants in comparison with other food categories, although it was lower among participants aged 12–19 years (Table 3). Milk intake was particularly important among Anishinabe participants aged 6–11 years (median 592 g/d) and Innu participants aged 3–5 and 6–11 years (medians 590 and 607 g/d, respectively). Participants drank almost as much milk as other beverages, including water. Canned foods were consumed more by Anishinabe participants, whereas desserts were consumed more among Innu participants. Ultra-processed food intake was higher among participants aged 12–19 years among both nations (105 g/d for the Anishinabe participants; 179 g/d for the Innu participants), with no statistical difference between the two nations.

**Table 3:** Median (510^th^–95^th^ percentile) of food intake by food category or item (g/d), for JES!-YEH! participants by nation and age groups

	Anishinabe								Innu							
	Total (*n* 107)		3–5 years (*n* 23)		6–11 years (*n* 45)		12–19 years (*n* 39)		Total (*n* 78)		3–5 years (*n* 13)		6–11 years (*n* 28)		12–19 years (*n* 37)	
	Median	10^th^–95^th^ percentile	Median	10^th^–95^th^ percentile	Median	10^th^–95^th^ percentile	Median	10^th^–95^th^ percentile	Median	10^th^–95^th^ percentile	Median	10^th^–95^th^ percentile	Median	10^th^–95^th^ percentile	Median	10^th^–95^th^ percentile
Market foods																
Any dairy product	665†	263–1615	693	221–1615	743	362–1425	461	243–1757	544	213–1493	870	317–1556	699	274–1556	322	210–1338
Milk	472*	203–1180	337	118–1072	592	236–1180	288	140–1202	272	101–1163	590	156–1163	607	118–1167	203	101–713
Yoghurt	71	12–413	130	71–413	130	23–413	71	0–413	71	12–498	130	71–743	71	71–413	71	0–498
Cheese	32	8–164	48	16–175	32	8–142	28	8–164	48	8–196	48	16–196	40	12–88	32	8–263
Eggs	20	2–94	13	2–118	13	0–47	40	10–94	20	3–176	13	3–31	13	4–37	40	3–235
Meat	83	25–249	53	22–153	69	29–162	103	25–284	76	35–264	59	19–201	61	31–136	103	50–284
Processed meat	12	2–38	4	2–19	13	0–35	12	6–66	12	2–38	6	2–19	6	2–19	12	6–88
Fruit and vegetables	345*	135–756	312	135–711	281	120–580	398	167–916	279	80–749	270	141–515	255	106–456	300	55–1308
Fat and oil	8	2–30	5	1–15	7	1–17	16	7–49	11	3–44	6	3–20	7	1–24	16	5–53
Beverages	968†	414–3050	536	221–1522	904	414–1830	1379	716–4384	870	182–3248	333	125–1565	556	140–1831	1435	292–4818
Ultra-processed food	83	25–274	33	21–168	61	23–191	105	46–359	79	22–284	76	30–137	49	20–198	179	37–376
Starch products	391	181–757	271	181–518	378	171–565	486	315–841	408	205–809	292	183–633	312	197–615	490	281–826
Canned foods	113**	31–284	112	23–217	99	26–253	122	46–426	69	19–221	63	19–314	69	23–196	76	7–221
Dessert	44	21–167	36	18–150	40	20–124	61	32–336	103***	41–269	94	38–217	76	37–215	146	51–443
Popcorn	2	0–7	1	0–5	2	0–5	2	0–10	1	0–10	1	0–5	1	0–5	2	0–10
Traditional foods																
Terrestrial meat	5·8***	0–114·1	3·9	0–155·2	5·8	0–35·7	11·6	0–160·6	0	0–30·1	0	0–6·9	0	0–18·7	0	0–71·4
Wild bird	0	0–35·8	0	0–24·7	0	0–12·0	0	0–47·6	0	0–43·1	0	0–23·2	1·9	0–23·5	0	0–45·9
Fish	0	0–72	0	0–37	0	0–25	0	0–171	12·0***	0–86	4·0	0–74	10·5	0–80	23·9	0–257
Seafood	0	0–0	0	0–0	0	0–0	0	0–0	0***	0–57·8	0	0–4·6	0	0–8·2	0	0–97·5
Wild fruit	0	0–17·7	0	0–14·7	0	0–7·4	0	0–35·4	9·3***	0–168·1	4·9	0–128·5	6·1	0–188·7	14·7	0–168·1

* *P* < 0·05.

† *P* ≤ 0·10.

** *P* < 0·01.

*** *P* < 0·001.

Food intakes (g/d): median (10^th^–95^th^ percentile).

Significant difference between age group for all participants assessed using Kruskall–Wallis test, the age category with different letter = *P* < 0·05.

Significant difference between participants from Anishinabe and Innu communities assessed using Wilcoxon test.

For of the list of food items included in each food category, please refer to the supplemental Table S4 and Table S5.

Overall traditional food intake was lower than market foods and consumption patterns were dependent on surrounding systems, i.e. coastal or terrestrial ecosystems (Table 3). Anishinabe more often consumed terrestrial meat (moose, hare and beaver), whereas Innu participants consumed wild-caught fish (salmon, trout, cod and American smelt) (see online Supplemental Table S5), and their intake both increased with age. At the time of the study, Innu participants also consumed a variety of wild local berries (primarily, blueberries, raspberries and strawberries, but also included cloudberries and redberries), which also increased with age. Wild bird and other seafood intake was marginal.

All dairy products (milk, yoghurt, cheese) were associated with serum PFNA levels among Anishinabe and Innu participants (GM ratio: 1·53 (95 % CI 1·03, 2·29) and 1·52 (95 % CI 1·05, 2·20) respectively) (Table 4). Among Anishinabe participants, PFNA was also positively associated with ultra-processed foods (GM ratio: 1·57 (95 % CI 1·07, 2·31)) and suggestively associated with starch products (GM ratio: 1·48 (95 % CI 0·95, 2·31)), while among Innu participants, PFNA was also associated with intake of wild fish (GM ratio: 1·44 (95 % CI 1·07, 1·94)) and wild berries (GM ratio: 1·75 (95 % CI 1·30, 2·36)) (Table 4). Positive associations were observed between serum PFOA and dairy products (GM ratio: 1·14 (95 % CI 0·98, 1·32)), desserts (GM ratio: 1·18 (95 % CI 0·98, 1·41)) and wild meat intake (GM ratio: 1·11 (95 % CI 0·99, 1·25)) among Anishinabe participants, but none reached statistical significance. A positive association between serum PFHxS and popcorn intake was also observed (GM ratio: 1·31 (95 % CI 1·04, 1·65)) among Anishinabe participants (Table 5). No associations between PFOS and food products were observed.

**Table 4:** Adjusted geometric mean (GM) of PFNA and PFOA (µg/l) and the adjusted chemical GM ratio by food category/items, stratified by nation

	PFNA						PFOA					
	Anishinabe (*n* 107)			Innu (*n* 78)			Anishinabe (*n* 107)			Innu (*n* 78)		
	GM	GM ratio	95 % CI	GM	GM ratio	95 % CI	GM	GM ratio	95 % CI	GM	GM ratio	95 % CI
Dairy products												
Low	4·06			0·53			0·82			0·79		
High	6·23	1·53*	1·03, 2·29	0·81	1·52*	1·05, 2·20	0·93	1·14†	0·98, 1·32	0·84	1·06	0·95, 1·19
Milk												
Low	4·1			0·57			0·83			0·8		
High	6·26	1·53*	1·05, 2·23	0·74	1·29	0·90, 1·86	0·92	1·10	0·95, 1·28	0·83	1·04	0·93, 1·15
Yoghurt												
Low	4·84			0·61			0·89			0·83		
High	5·43	1·12	0·78, 1·62	0·7	1·14	0·81, 1·60	0·86	0·97	0·84, 1·12	0·78	0·94	0·83, 1·05
Cheese												
Low	4·66			0·68			0·93			0·81		
High	5·67	1·22	0·86, 1·73	0·61	0·90	0·67, 1·21	0·82	0·88	0·74, 1·04	0·81	1·01	0·89, 1·13
Processed meat												
Low	4·64			0·71			0·84			0·8		
High	5·55	1·17	0·85, 1·61	0·58	0·82	0·56, 1·21	0·9	1·07	0·93, 1·25	0·82	1·02	0·90, 1·15
Ultra-processed food												
Low	4·07			0·66			0·88			0·82		
High	6·41	1·57*	1·07, 2·31	0·62	0·95	0·65, 1·39	0·87	0·99	0·86, 1·15	0·8	0·96	0·86, 1·08
Starch product												
Low	4·21			0·67			0·84			0·88		
High	6·24	1·48†	0·95, 2·31	0·62	0·93	0·63, 1·36	0·91	1·08	0·93, 1·24	0·75	0·84	0·73, 0·97
Popcorn												
Low	5·07			0·63			0·89			0·81		
High	5·19	1·02	0·71, 1·47	0·67	1·07	0·75, 1·52	0·84	0·95	0·81, 1·11	0·81	0·9	0, 1·11
Dessert												
Low	4·88			0·57			0·83			0·76		
High	5·64	1·16	0·84, 1·59	0·67	1·16	0·80, 1·68	0·98	1·18†	0·98, 1·41	0·83	1·09	0·96, 1·24
Traditional food: terrestrial meat												
Low	5·2			–			0·81			–		
High	5·07	0·98	0·67, 1·41	–			0·91	1·11†	0·99, 1·25	–		
Traditional food: fish												
Low	–			0·51			–			0·8		
High	–			0·73	1·44*	1·07, 1·94	–			0·81	1·02	0·91, 1·14
Traditional food: wild fruit												
Low	–			0·46			–			0·81		
High	–			0·8	1·75**	1·30, 2·36	–			0·81	1·00	0·89, 1·13

PFNA, perfluorononanoic acid; PFOA, perfluorooctanoic acid.

Significant GM ratio.

* *P* < 0·05.

† *P* ≤ 0 10.

** *P* < 0·001.

Adjusted for sex, age and age-squared low and high food categories were dichotomised according to the median of participant’s consumption in g/d: low (≤ median) and high (> median). If median = 0, the analyses were not processed. For the list of food items included in each food category, please refer to the Supplemental Tables S4 and S5.

**Table 5:** Adjusted geometric mean (GM) of PFHxS and PFOS (µg/l) and BPA (μg/g creatinine) and the adjusted chemical GM ratio by food category/items, stratified by nation

	PFHxS						PFOS					
	Anishinabe (*n* 107)			Innu (*n* 78)			Anishinabe (*n* 107)			Innu (*n* 78)		
	GM‡	GM‡ ratio	95 % CI	GM‡	GM‡ ratio	95 % CI	GM	GM ratio	95 % CI	GM	GM ratio	95 % CI
Dairy products												
Low	0·53			0·24			1·11			0·91		
High	0·53	1·00	0·78, 1·28	0·26	1·09	0·88, 1·35	0·96	0·87	0·71, 1·06	1·14	1·24	0·99, 1·56
Milk												
Low	0·54			0·23			1·13			0·93		
High	0·52	0·95	0·76, 1·19	0·27	1·20	0·96, 1·50	0·94	0·83	0·68, 1·01	1·09	1·17	0·92, 1·50
Yoghurt												
Low	0·52			0·26			1·09			1·06		
High	0·54	1·03	0·82, 1·29	0·23	0·89	0·72, 1·11	0·97	0·89	0·73, 1·08	0·92	0·87	0·69, 1·10
Cheese												
Low	0·53			0·22			1·12			0·79		
High	0·53	1·00	0·81, 1·23	0·27	1·20	0·96, 1·51	0·94	0·84	0·71, 1·01	0·92	1·11	0·89, 1·38
Processed meat												
Low	0·54			0·26			1·04			1·04		
High	0·52	0·98	0·78, 1·23	0·23	0·90	0·74, 1·10	1·02	0·98	0·80, 1·20	0·98	0·94	0·74, 1·19
Ultra-processed food												
Low	0·56			0·25			1·11			1·08		
High	0·5	0·89	0·71, 1·12	0·24	0·95	0·76, 1·20	0·96	0·87	0·72, 1·05	0·93	0·86	0·67, 1·09
Starch product												
Low	0·53			0·25			0·94			1·07		
High	0·53	1·01	0·83, 1·23	0·24	0·97	0·75, 1·26	0·87	0·92	0·77, 1·10	0·95	0·89	0·67, 1·18
Popcorn												
Low	0·48			0·24			1·07			0·99		
High	0·63	1·31*	1·04, 1·65	0·27	1·14	0·86, 1·52	0·96	0·89	0·74, 1·09	1·04	1·05	0·84, 1·33
Dessert												
Low	0·53			0·23			0·97			1·02		
High	0·53	1·02	0·83, 1·24	0·25	1·11	0·89, 1·38	1·16	1·19	0·96, 1·47		0·98	0·77, 1·25
Traditional food: terrestrial meat												
Low	0·54			–			0·96			–		
High	0·52	0·97	0·78, 1·21	–			1·07	1·11	0·91, 1·35	–		
Traditional food: fish												
Low	–			0·23			–			0·92		
High	–			0·25	1·08	0·88, 1·33	–			1·06	1·16	0·95, 1·40
Traditional food: wild fruit												
Low	–			0·27			–			1·08		
High	–			0·23	0·88	0·70, 1·10	–			0·96	0·89	0·71, 1·12

PFHxS, perfluorohexane sulphonic acid; PFOS, perfluorooctanesulfonic acid; BPA, bisphenol-A.

Significant geometric mean ratio.

* *P* < 0·05.

‡ Adjusted for sex, age and age-squared low and high food categories were dichotomised according to the median of participant’s consumption in g/d: low (≤ median) and high (> median).

If median = 0, the analyses were not processed. For of the list of food items included in each food category, please refer to the Supplemental Tables S4 and S5.

For urinary BPA, positive associations were observed with the consumption of cheese (GM ratio: 1·72 (95 % CI 1·19, 2·50)) and milk (GM ratio: 1·53 (95 % CI 1·02, 2·29)) among Anishinabe participants, and desserts (GM ratio: 1·71 (95 % CI 1·07, 2·74)), processed meats (GM ratio: 1·55 (95 % CI 1·00, 2·38)), wild fish (GM ratio: 1·64 (1·07, 2·49)) and wild berries (GM ratio: 2·06 (95 % CI 1·37, 3·10)) amongst Innu participants (Table 6).

**Table 6:** Adjusted geometric mean (GM) of BPA (μg/g creatinine) and the adjusted chemical GM ratio by food category/items, stratified by nation

	BPA					
	Anishinabe (*n* 107)			Innu (*n* 78)		
	GM‡	GM‡ ratio	95 % CI	GM‡	GM‡ ratio	95 % CI
Dairy products						
Low	1·39			1·53		
High	2·03	1·47	0·95, 2·25	1·64	1·08	0·56, 2·07
Milk						
Low	1·37			1·54		
High	2·09	1·53*	1·02, 2·29	1·63	1·06	0·58, 1·93
Yoghurt						
Low	1·43			1·45		
High	2·06	1·44	0·96, 2·16	1·82	1·26	0·74, 2·14
Cheese						
Low	1·32			1·34		
High	2·27	1·72*	1·19, 2·50	1·82	1·36	0·88, 2·09
Processed meat						
Low	1·62			1·26		
High	1·75	1·08	0·73, 1·60	1·81	1·55*	1·00, 2·38
Ultra-processed food						
Low	1·63			1·44		
High	1·77	1·08	0·69, 1·70	1·73	1·20	0·78, 1·85
Starch product						
Low	1·64			1·36		
High	1·77	1·08	0·61, 1·90	1·81	1·33	0·84, 2·09
Popcorn						
Low	1·7			1·48		
High	1·7	1·00	0·66, 1·52	1·87	1·27	0·70, 2·29
Dessert						
Low	1·67			1·06		
High	1·77	1·06	0·62, 1·82	1·81	1·71*	1·07, 2·74
Traditional food: terrestrial meat						
Low	0·81			–		
High	0·91	1·11	0·99, 1·25	–		
Traditional food: fish						
Low	–			1·15		
High	–			1·88	1·64*	1·07, 2·49
Traditional food: wild fruit						
Low	–			1·02		
High	–			2·1	2·06**	1·37, 3·10

BPA, bisphenol-A.

Significant GM ratio.

* *P* < 0·05.

** *P* < 0·001.

Adjusted for sex, age and age-squared low and High food categories were dichotomised according to the median of participant’s consumption in g/d: low (≤ median) and high (> median).

If median = 0, the analyses were not processed. For of the list of food items included in each food category, please refer to the Supplemental Tables S4 and S5.

As shown in Table 7, after removing Anishinabe participants aged 6–11 years, despite the high magnitudes in the GM ratios, the associations between dairy products (GM ratio: 1·35 (95 % CI 0·75, 2·42); *P*-value 0·32) and milk (GM ratio: 1·43 (95 % CI 0·84, 2·42); *P*-value 0·19) and serum PFNA were no longer significant. However, the associations with ultra-processed foods remained significant (GM ratio: 1·98 (95 % CI 1·13, 3·47)) and starch products became significant (GM ratio: 1·90 (95 % CI 1·00, 3·63)).

**Table 7:** Adjusted geometric mean (GM) of PFNA (µg/l) and adjusted GM ratio by food category/items significantly associated with PFNA serum levels in multiple linear regression models for Anishinabe JES!-YEH participants without those aged 6–11 years (*n* 62)

	Anishinabe (*n* 62)		
	GM‡	GM‡ ratio	95 % CI
Traditional food: terrestrial meat			
Low	3·67		
High	3·11	0·85	0·50, 1·44
Dairy products			
Low	2·89		
High	3·90	1·35	0·75, 2·42
Milk			
Low	2·83		
High	4·03	1·43	0·84, 2·42
Yoghurt			
Low	2·66		
High	4·23	1·59	0·90, 2·79
Cheese			
Low	2·80		
High	3·92	1·40	0·77, 2·53
Processed meat			
Low	3·28		
High	3·28	1·00	0·62, 1·63
Ultra-processed food			
Low	2·21		
High	4·37	1·98	1·13, 3·47*
Starch product			
Low	2·30		
High	4·39	1·90	1·00, 3·63*
Popcorn			
Low	3·17		
High	3·52	1·11	0·64, 1·94
Dessert			
Low	2·96		
High	3·91	1·32	0·82, 2·13

PFNA, perfluorononanoic acid.

Significant GM ratio.

* *P* < 0·05.

‡ Adjusted for sex, age and age-squared.

Adjusted for sex, age and age-squared.

Low and High food categories were dichotomised according to the median of participant’s consumption in g/d: low, (≤ median) and high (> median). If median = 0, the analyses were not processed.

For of the list of food items included in each food category, please refer to the Supplemental Tables S4 and S5.

## Discussion

We conducted a cross-sectional study in youth aged 3–19 years from Anishinabe and Innu communities in northern Quebec to compare serum PFAA and urinary BPA biomarkers across nations and the general Canadian population, and to study the association between food category/item consumption and the chemical exposures. Serum PFNA levels were higher in Anishinabe *v*. Innu participants and the CHMS Cycle 5; however, PFOS, PFOA and PFHxS were generally higher in CHMS Cycle 5 or on par with Anishinabe and Innu participants. BPA was higher among Anishinabe and Innu participants aged 12–19 years compared with CHMS Cycle 5. Associations between foods and chemical levels differed by nation, explained in part by different consumption patterns across nations. In general, among Anishinabe participants, different categories of market foods were associated with serum PFNA, PFOA, PFHxS and urinary BPA, whereas among Innu, only serum PFNA and urinary BPA were associated with market foods. Although local traditional foods were not initially identified as a potential source of exposure, significant associations were observed between wild fish and berry consumption and PFNA and BPA exposures among Innu participants, and a similar trend was observed for wild meat and PFOA and BPA exposures among Anishinabe participants.

### Levels of exposure to PFAA and BPA in the JES!-YEH! Study

The serum PFNA concentrations measured in Anishinabe participants (GM: 5·12 µg/l) were also markedly higher when compared with PFNA concentrations in children in the USA. For example, the GM of PFNA serum concentrations was 1·7 µg/l in children aged 6–10 years living in the Boston area who participated in the Viva project (2007–2010)^(44)^. In children under 12 years participating in the PEASE project in New Hampshire, the GM of serum PFNA concentration was 0·92 µg/l in 2015–2016^(45)^. Finally, the PFNA mean serum concentration in the National Health and Nutrition Examination Survey (NHANES) survey (2013–2014) across the USA for children aged 3–11 years was 0·79 µg/l^(45)^.

PFAA remain detected ubiquitously in the environment and consumer products due to their environmental persistence, their continued production in some countries, and the degradation of other fluorinated chemicals like fluorotelomer alcohols into PFAA compounds^(7,15,46,47)^. Due to the high levels of PFNA in the Anishinabe participants, several potential local sources were investigated by the research team and partners. Municipal water tests showed no detection of PFNA in the community’s drinking water. Furthermore, several community members drink bottled water. Several other possible sources of PFNA were tested (cleaning products, floor waxes, furniture, carpets, etc.) but no products that could contain these substances were identified at the elementary school, by the band council or in the participant’s residences^(34)^. Other potential dietary sources remain to be investigated.

In our study, urinary BPA concentrations were higher among Anishinabe and Innu participants aged 12–19 years (GM: 2·29 and 1·66 µg/g creatinine, respectively) compared with similar age groups in CHMS Cycle 5. These were, however, lower compared with urinary BPA concentrations in NHANES (2003–2004), with GM 4·3 and 2·8 µg/g creatinine among children aged 6–11 and 12–19 years old, respectively^(48)^.

In recent years, the Government of Canada examined the health effects of BPA and concluded that exposure levels in the Canadian population are below those that could cause health effects, but remained concerned regarding potential health implications of chronic exposure to low doses of BPA^(49)^. Since 2010, actions were taken to further protect newborns and infants by prohibiting the manufacture, import, advertising and sale of baby bottles containing BPA^(49)^. In addition, since 2014, the Government of Canada has been prohibiting the use of packaging with BPA for liquid infant formula^(49)^. No other systemic actions were taken to remove BPA from other market foods consumed by children and youth.

### Diet characteristics in the JES!-YEH! Study

In the JES!-YEH! project, dairy products were widely consumed among Anishinabe and Innu participants in comparison with other food categories, particularly with regards to milk among Anishinabe aged 6–11 years and Innu aged 3–5 years. A Health Report by Statistics Canada reported that children aged 1–8 years consumed an average of 360–366 g/d and children aged 9–13 years consumed 288–364 g/d of different types of milk in 2015^(50)^. The consumption of milk in Anishinabe children aged 6–11 years (592 g/d) exceed milk consumption in both age groups from the Health Report. The intake of ultra-processed products (chips, French fries, poutine and pizza) was also elevated in our study population and increased with age. Similarly, desserts (ice cream, pies, candy bars, etc.) increased with age, but were more heavily consumed among Innu compared with Anishinabe participants. Popcorn and processed meats were consumed in similar quantities by both nations.

The overall intake of market products was much higher than traditional foods, and these results corroborate other studies highlighting that intake of market food is continuously replacing traditional food in many Indigenous youth populations^(1,3,51–53)^. Several studies highlight that although traditional diets have become increasingly marginal in some communities, it remains a key source of nutrients and plays a central role for the transmission of Indigenous culture^(51,53)^. Moreover, interventions promoting traditional activities have been shown to be very successful for children and youth health, well-being and resilience^(1)^.

### Diet and exposure to contaminants in the JES!-YEH! Study

Serum levels of PFNA were associated with the intake of dairy products (participants from both nations) and milk (Anishinabe participants), albeit the association in Anishinabe was no longer significant after removal of those aged 6–11 years. BPA serum levels were associated with the intake of cheese and milk among Anishinabe participants. These results are consistent with several other studies which reported the presence of PFAA and BPA in dairy samples^(6,8,18,22,25,54–56)^. Two other epidemiological studies in the USA conducted in adults and children also found positive associations between intake of dairy products and exposure to PFAA^(7,43)^, and several studies specifically detected PFNA and BPA in commercial milk samples^(22,57–60)^. Even though many authors have reported the occurrence of contaminants in milk, data on contamination pathways along the dairy chain are limited. Oil-resistant packaging containing PFAA may explain the detection of PFAA in dairy products as PFAA present a great affinity for proteins^(7)^. Other studies have also speculated that PFAA could accumulate in field crop plants and further bioaccumulate in livestock, which could lead to elevated PFAA in milk^(61,62)^. Some authors also argue that BPA may be found in the equipment used to produce milk in processing plants (plastic tube, sealer, etc.) or the milk storage tank. Alternatively, there may be direct contamination from the farm through ingestion of contaminated feed or via the uptake of chemical compounds as a result of cows grazing on contaminated soils^(25,63–65)^. Thus, BPA local contamination could lead to elevated BPA in the milk chain as BPA residues bioaccumulate in cow adipose tissue, eventually getting secreted in milk fat and accumulated in fatty dairy products^(59,66)^.

The intake of processed food (e.g. ultra-processed foods, popcorn, processed meat and desserts) was also associated with serum levels of PFNA, PFHxS and/or urinary level of BPA. Migration of PFAA from food packaging may increase when the food packaging is subjected to high temperatures (such as microwave popcorn), when it is in prolonged contact with the food and when emulsifiers are present in the packaging^(22,67)^. More specifically, PFNA has been detected in various packaging used for ultra-processed foods (baking dishes, fast-food packaging, muffin packaging and microwavable popcorn bags)^(68–70)^, and appears to migrate to foods from packaging in highly fatty and alcoholic foods^(71,72)^. Indeed, several studies detected PFAA or BPA in ultra-processed products such as pastries, microwaveable popcorn, pizza, french fries, hot dogs, etc^(7,8,43,73,74)^. Conversely, contrary to several studies that have found PFAA or BPA in canned food^(6,7,22,24,54,55)^, we did not detect any associations between the intake of canned food items and serum levels of these contaminants. BPA coating is not consistently found in canned food (metal canning rods, epoxy resin of the rods, Mason jars and aluminium cane)^(19,21,22)^, and very often, it depends on the brand of the product purchased. Fortunately, BPA is progressively being removed from canned foods (such as Mason jar lids whose seals are now BPA-fee). This, however, makes the use of FFQ to capture BPA exposure via canned foods difficult since most consumers do not notice the presence or absence of BPA coatings. In the present study, we only assessed the overall intake of canned food and this may also have diluted the association between canned food intake and BPA exposure.

Our analyses revealed that among the Innu participants in JES!-YEH!, the intake of some traditional foods (e.g. wild fish and berries) was associated with serum levels of PFNA and urinary levels of BPA. A weak association was also found between terrestrial meat intake and serum PFOA. A few studies have reported the presence of PFAA and BPA in market fish, meat and fruits^(7,11,42,43,54–57,74,75)^. Conversely, another study reported low levels of PFAA in traditional foods from the two studied nations^(1)^.

Two hypotheses can be raised to explain the presence of these contaminants in traditional foods. First, following hunting, fishing and gathering, traditional foods are usually preserved in plastic bags (i.e. grocery bags or Ziplock® bags) or disposable plastic containers usually made of polycarbonate plastic (i.e. hard plastic containers), and sometimes, for an extended period of time in the freezer or canned in Mason jars, which would explain the presence of BPA. Second, PFAA and BPA contamination of the environment (soil, air, water) and wildlife (terrestrial and aquatic organisms) has been documented^(47,76,77)^. In particular, recent studies have documented high levels of PFNA and other long-chain PFAA compared with other persistent organic pollutants in moose liver, marine mammals, fish and caribou in the Canadian Arctic. Long-chain PFAA are also known to be on the rise in Inuit populations in Nunavik, Northern Quebec^(34,78,79)^. Conversely, no local source of these contaminants could be identified in these boreal ecosystems, and the source of these chemicals would likely be a result of long-range atmospheric and oceanic transportation^(80,81)^, as it is the case in the Arctic. Moreover, PFAA concentrations were found in higher concentrations in terrestrial and freshwater biota *v*. marine biota^(79,82,83)^. Anishinabe communities are more likely to consume freshwater seafood, whereas Innu communities are more likely to eat marine seafood. This may also explain the elevated concentrations of PFNA in Anishinabe *v*. Innu participants.

Contrary to PFAA that are very persistent and travel over long distances, BPA is rapidly degraded in the environment under aerobic conditions. Indeed, BPA half-lives range from 2 to 3 d in river water^(84)^, and less than 3 d in soil^(85)^. Thus, is it is unlikely that BPA could accumulate in traditional foods in these remote regions.

### Limitations

The recruitment of First Nations children was limited by the community sizes involved in the study, resulting in a relatively smaller sample size. Differential information biases may have been introduced into the dietary questionnaire results since the parent or guardian answering the dietary questionnaires may not necessarily be present at all meals in the child’s daily routine (e.g. lunch at school or daycare, purchase of additional food in stores). In addition, parents may have responded according to norms of social acceptability, especially when it came to the consumption of ultra-processed foods. This could result in an underestimation of the frequency of intake of low-nutrient foods and an overestimation of nutritious foods. Nevertheless, parents were identified as being in the best position to provide information about their children’s diets. We were unable to account for specific food types/brands, food supply, breastfeeding/in-utero exposures and socio-economic status. Finally, it is important to note that the children were not fasting when the samples were taken and that participants provided only one spot urine sample. Considering the short half-life of BPA, further studies should consider administering multiple urine samples at different points in time, as well as include a longer analyte list that considers other long-chain PFAA congeners that have been detected in Circumpolar and northern regions. However, these results raise important hypotheses that deserve to be further investigated with particular attention to traditional *v*. market foods, as well as comparisons of Indigenous and non-Indigenous children and youth.

## Conclusion

Our results highlight that diets with more highly processed foods and dairy products in the studied communities were associated with exposure to PFNA and BPA. Mean PFNA serum levels were significantly higher in JES!-YEH! Anishnabe participants compared with CHMS Cycle 5 (2016–2017), while mean urinary BPA levels were higher in older JES!-YEH! participants compared with CHMS cycle 5. Moreover, this disproportionate exposure could potentially contribute to the increased prevalence of cardiometabolic diseases and developmental outcomes among Indigenous youth. These findings highlight the importance of better documenting food processing and packaging methods, and increased measurement of these chemicals in dairy and traditional foods before and after processing and packaging. Furthermore, these results underscore the importance of promoting the consumption of minimally processed and unpackaged foods to provide healthier food environments for youth in Indigenous communities and beyond.

## Supplementary material

For supplementary material accompanying this paper visit https://doi.org/10.1017/S1368980022000581. click here to view supplementary material
