# Supplementary material for: Perfluoroalkyl acid and bisphenol-A exposure via food sources in four First Nation communities in Quebec, Canada
Source: Public Health Nutr. 2022 Mar 11;26(1):106–21. doi: 10.1017/S1368980022000581 (PMC11077462; doi:10.1017/S1368980022000581)
Supplement: Supplementary file 1 [file S1368980022000581sup001.docx]

**Supplementary tables for “Perfluoroalkyl acid and bisphenol-A exposure via food sources in four First Nation communities in Quebec, Canada”**

Table S1: Literature review of foods with detected PFAAs measured in food samples

Table S2: Literature review of foods with detectable PFAAs (associative studies)

Table S3: Literature review of foods with detectable BPA measured in food samples

Table S4: Detailed description of market food categories and items

Table S5: Detailed description of traditional food categories and items

Table S6: Distributions of serum concentrations of perfluorononanoic acid (PFNA), perfluorooctane sulfonate (PFOS), perfluorooctanoic acid (PFOA) and perfluorohexane sulfonate (PFHxS) (µg/L) and urine concentrations of bisphenol A (BPA) (μg/g creatinine) in participants from JES!-YEH! (2015) by nation

Table S7: Proportion of JES!-YEH! Participants with exposure levels above 95th percentiles (P95) for perfluoroalkyl substances in plasma and bisphenol A in urine for the Canadian Health Measure Suvey Cycle 5 (2016-2017)

Table S1: Literature review of foods with detected PFAAs measured in food samples

| **Study** | **Year of sample collection** | **Food** | **PFAA detected** |
| --- | --- | --- | --- |
| Domingo et al., 2012^(1)^ | 2011 | Fish and shellfish | PFOS, PFOA, PFHpA, PFHxS, PFDS |
|  |  | Lettuce | PFOA, PFHpA, PFHxS |
|  |  | Carrot | PFOA |
|  |  | Dairy | PFOA, PFHpA |
|  |  | Eggs | PFHpA |
|  |  | Ham | PFHxS |
|  |  | Sausage | PFHxS |
|  |  | Pasta | PFDS |
|  |  | Seafood | PFDA |
|  |  | Potatoes | PFDA |
|  |  | Apple | PFDA |
|  |  | Orange | PFDA |
|  |  | Canned product | PFDA, PFHxS, PFDS |
| Eriksson et al., 2013^(2)^ | 2012 | Milk | PFUnDA, PFNA |
|  |  | Fish | PFUnDA, PFOA, PFOS |
|  |  | Yoghurt | PFOS |
| Haug et al., 2010^(3)^ | 2008-2009 | Margarine | PFHxS, PFOS, PFHxA, PFOA |
|  |  | Bread | PFHxS, PFOS, PFHxA, PFOA, PFNA |
|  |  | Pork | PFHxS, PFOS, PFOA, PFDA |
|  |  | Egg | PFHxS, PFOS, PFHxA |
|  |  | Salmon | PFHxS, PFOS |
|  |  | Cod | PFHxS, PFOS |
|  |  | Fruits and vegetables | PFOS, PFHxA, PFOA, PFDA |
|  |  | Cheese | PFOS, PFNA |
|  |  | Milk | PFOS, PFHxA, PFOA, PFDA |
|  |  | Beef | PFOS, PFOA, PFNA |
|  |  | Chicken | PFOS, PFOA |
|  |  | Fishsticks | PFOS |
|  |  | Canned product | PFOS |
| Ostertag et al., 2009^(4)^ | 1998 | Cookies | PFOA, PFNA |
|  |  | Cheese | PFOA, PFOS |
|  |  | Peppers | PFOA |
|  |  | Pizza | PFOA |
|  |  | Cold cuts | PFNA |
| Tittlemier et al., 2007^(5)^ | 2004 | Beef | PFNA, PFOA, PFOS |
|  |  | Microwave popcorn | PFOA |
|  |  | Fish | PFOS |
| Vestergren et al., 2012^(6)^ | 2010 | Dairy | PFHxS, PFOA |
|  |  | Meat | PFOS. PFOA, PFDA, PFHxS |
|  |  | Fats | PFOS |
|  |  | Fish | PFOS, PFOA, PFNA, PFDA, PFHxS |
|  |  | Egg | PFOA, PFOS |
|  |  | Cereal | PFOA |
|  |  | Vegetables (including root vegetables) | PFOA, PFOS |
|  |  | Fruits | PFOA |
| Wang et al., 2010^(7)^ | 2008-2009 | Milk | PFNA, PFHpA |
|  |  | Yoghurt | PFOA |

Table S2: Literature review of foods with detectable PFAAs (associative studies)

| **Study** | **Population** | **Year of sample collection** | **Food item** | **Chemical detected** | **Association measure (95% CI)** | **Covariates** |
| --- | --- | --- | --- | --- | --- | --- |
| Halldorsson et al., 2008^(8)^ | 1076 pregnant women | 1996 - 2002 | Dietary variables with food frequency questionnaire at mid-pregnancy, quartile categories | Plasma level of PFOS (ng/mL) | Mean  difference in plasma PFOS levels (ng/mL) from the lowest to the highest intake quartile in food intake (CI 95%) | Parity, smoking, maternal age, pre-pregnancy BMI, socio-occupational status |
|  |  |  | Red meat | PFOS | 4.3 (2.1-6.5) |  |
|  |  |  | Animal fats | PFOS | 3.4 (1.2-5.6) |  |
|  |  |  | Snacks | PFOS | 2.0 (0.3- 3.6) |  |
| Wu et al., 2015^(9)^ |  | 2007 -2009 | Frequency of eating (time/week) with food frequency questionnaire,  Continuous variable | Serum sample of PFAA (ng/mL) | Slope (SE)  p-value |  |
|  | 149 adults from California |  | Margarine/butter | PFNA | 0.04 (0.04)  0.02 | Age, occupational exposure |
|  |  |  | Beef | PFOS | 0.08 (0.03)  0.03 |  |
|  |  |  | Canne Meat entrée | PFOS | 0.18 (0.07)  0.01 |  |
|  |  |  | Canned Meat entrée | PFHxS | 0.19 (0.08)  0.02 |  |
|  |  |  | Fresh tuna and white fish | PFHxS | 0.17 (0.08)  0.05 |  |
|  |  |  | Freshwater fish | PFOS | 0.50 (0.14)  0.00 |  |
|  | 68 children from California |  | Hot-dog | PFOS | 0.22 (0.08)  0.01 | Age, frequency of wearing waterproof clothes |
|  |  |  | French fries | PFNA | 0.17 (0.08)  0.05 |  |
|  |  |  | Chips | PFOA | 0.07 (0.03)  0.01 |  |
| Park et al., 2019^(10)^ | 1302 women aged 45-56 years old | 16 follow-up visit annually or biannually from 1996-1997 to 2016-2017 | Tertile of food consumption | Plasma level of PFAA | Unadjusted geometric mean p-value | Unadjusted |
|  |  |  | Fish  Tertile 1  Tertile 2  Tertile 3  p-value | PFNA | 0.51 (0.48, 0.53)  0.54 (0.51, 0.56)  0.61 (0.57, 0.65)  <0.0001 |  |
|  |  |  | Dairy  Tertile 1  Tertile 2  Tertile 3  p-value | PFOA | 3.88 (3.65, 4.12) 4.08 (3.87, 4.30) 4.26 (4.05, 4.48)  0.01 |  |
|  |  |  | Dairy  Tertile 1  Tertile 2  Tertile 3  p-value | PFHxS | 1.37 (1.27, 1.48) 1.61 (1.50, 1.73) 1.78 (1.65, 1.93)  <0.0001 |  |
|  |  |  | Pizza  Tertile 1  Tertile 2  Tertile 3  p-value | PFOA | 3.64 (3.37, 3.93) 4.01 (3.79, 4.24) 4.32 (4.14, 4.52)  <0.0001 |  |
|  |  |  | Pizza  Tertile 1  Tertile 2  Tertile 3  p-value | PFOS | 6.62 (6.05, 7.25) 7.12 (6.70, 7.58) 7.59 (7.19, 8.02)  0.005 |  |
|  |  |  | Salty snacks  Tertile 1  Tertile 2  Tertile 3  p-value | PFOA | 3.18 (2.94, 3.44) 3.94 (3.71, 4.18) 4.51 (4.33, 4.70)  <0.0001 |  |
|  |  |  | Salty snacks  Tertile 1  Tertile 2  Tertile 3  p-value | PFOS | 21.59 (20.08, 23.20) 24.15 (22.78, 25.60) 27.76 (26.59, 28.99)  <0.0001 |  |
|  |  |  | French fries  Tertile 1  Tertile 2  Tertile 3  p-value | PFOA | 3.68 (3.46, 3.92) 4.12 (3.83, 4.43) 4.28 (4.10, 4.46)  0.0005 |  |
|  |  |  | French fries  Tertile 1  Tertile 2  Tertile 3  p-value | PFOS | 6.47 (6.02, 6.95)  7.26 (6.70, 7.87)  7.67 (7.27, 8.08)  0.0006 |  |

Table S3: Literature review of foods with detectable BPA measured in food samples

| **Study** | **Sample year of collection** | **Food with BPA detected** |
| --- | --- | --- |
| Cao et al., 2011^(12)^ | 2008 | Baby food in jar and canned, cheese, butter, canned evaporated milk, processed meat, canned meat, poultry, canned soup, bread, cereal, canned vegetables, canned fruits, fast food |
| Cao et al., 2019^(13)^ | 2016 | Evaporated milk canned, soup canned, fresh beans, canned beans, fresh beets, canned beets, frozen corn, canned corn, frozen peas, canned peas, canned tomatoes |
| Grumetto et al., 2013^(15)^ | Not specified | Commercial milk |
| Santonicola et al., 2019^(14)^ | Not specified | Raw milk |
| Sungur et al., 2014^(11)^ | Not specified | Canned beans, canned corn, canned peas, canned tomato paste, canned tuna, boxed cream, boxed milk, boxed juice, boxed pudding, mushroom in glass jar, pickles in glass jar |

Table S4: Detailed description of market food categories and items

| **Market food category** | **Food item** | **Food item** |
| --- | --- | --- |
| **Nation** | **Anishinabe participants** | **Innu participants** |
| Canned food | Canned fruits  Canned milk  Tomato  Beans  Soup  Sardine  White tuna  Light tuna | |
| Dairy product | Milk (milk, chocolate milk, milk in coffee and milk formula*)  Yoghurt  Cheese (processed cheese and minimally processed) | Milk (milk, chocolate milk and milk in coffee)  Yoghurt  Cheese (processed cheese and minimally processed) |
| Dessert | Jam  White sugar  Ketchup*  Non-chocolate candies  Ice cream  Pie  Candy bars  Milk chocolate  Cake  Donuts  Sweeteners | Jam  White sugar  Non-chocolate candies  Ice cream  Pie  Candy bars  Milk chocolate  Cake  Donuts  Sweeteners  Granola bars*  Frozen desserts* |
| Drink | Bottled water  Powdered juices  Bottled juices or juice boxes  Regular carbonated beverages  Diet carbonated beverages  Tea  Sports drinks  Hot chocolate  Coffee  Energy drinks | Bottled water  Powdered juices  Bottled juices or juice boxes  Regular carbonated beverages  Diet carbonated beverages  Tea  Sports drinks  Hot chocolate  Coffee  Energy drinks  Herbal tea* |
| Fat and oil | Butter  Margarine  Mayonnaise  Frying oils (canola, olive, Crisco)  Vinaigrette | |
| Fish | Fresh or frozen fish  Sardine  Light tuna  White tuna | |
| Fruit and vegetable | Onion  Yellow vegetables  Other vegetables  Tomato  Green vegetables  Broccoli  Apple  Banana  Orange  Other fruits  Canned fruits  Store-bought berries | |
| Meat | Beef  Chicken  Pork  Hamburgers | |
| Processed meat | Sausage  Bacon  Chicken nuggets  Hot dogs  Beef jerky | |
| Starch product | White bread  Cold cereals  Pasta  Rice  Potatoes  Lipton soup  Crackers  Oven-baked bannock  Saco (fried bannock)*  Oatmeal  Brown bread | White bread  Cold cereals  Pasta  Rice  Potatoes  Lipton soup  Crackers  Oven-baked bannock  Oatmeal  Brown bread  Sand-baked bannock*  Tekaep (type of pancake/bannock with lingonberries)* |
| Ultra-processed food | Chips  Poutine  French fries  Pizza | |

*****Different food item in each region

Table S5: Detailed description of traditional food categories and items

| **Traditional food category** | **Food item** | **Food item** |
| --- | --- | --- |
| **Nation** | **Anishinabe participants** | **Innu participants** |
| Fish | Walleye*  Lake sturgeon*  Northern pike* | Atlantic salmon (boiled and smoked)*  Trout (brook)*  Atlantic cod*  Ouananiche (freshwater salmon)*  American smelt*  Lake trout* |
| Seafood | - | Lobster*  Scallop*  Crab* |
| Terrestrial meat | Moose (meat, liver, kidneys)  Hare  Beaver* | Moose (meat, liver, kidneys)  Hare  Caribou*  Castor* |
| Wild bird | Partridge  Goose  Spruce grouse* | Partridge  Goose  Moyak (common eider)*  American black duck*  Grouse* |
| Wild fruit | Blueberry  Raspberry  Strawberry | Blueberry  Raspberry  Strawberry  Lingonberry (redberry)*  Cloudberry* |

*****Different food item in each region

Table S6: Distributions of serum concentrations of perfluorononanoic acid (PFNA), perfluorooctane sulfonate (PFOS), perfluorooctanoic acid (PFOA) and perfluorohexane sulfonate (PFHxS) (µg/L) and urine concentrations of bisphenol A (BPA) (μg/g creatinine) in participants from JES!-YEH! (2015) by nation

|  | **Anishinabe (n= 107)** | | | | **Innu (n= 78)** | | | |
| --- | --- | --- | --- | --- | --- | --- | --- | --- |
|  | **GM (95% CI)** | **Median** | **Minimum** | **Maximum** | **GM (95% CI)** | **Median** | **Minimum** | **Maximum** |
| **PFNA** | 5.12* (4.28-6.11) | 6.25 | 0.42 | 29.00 | 0.64 (0.54-0.76) | 0.55 | 0.20 | 3.90 |
| **PFOS** | 1.03 (0.94-1.12) | 0.99 | 0.40 | 3.60 | 1.01 (0.91-1.11) | 0.97 | 0.40 | 5.90 |
| **PFOA** | 0.87 (0.81-0.94) | 0.86 | 0.18 | 2.90 | 0.81 (0.76-0.86) | 0.80 | 0.44 | 2.40 |
| **PFHxS** | 0.53* (0.48-0.59) | 0.49 | 0.21 | 2.80 | 0.25 (0.22-0.28) | 0.22 | 0.10 | 1.20 |
| **BPA** | 2.66 (2.18-3.25) | 2.30 | 0.41 | 67.32 | 1.90 (1.51-2.39) | 1.98 | 0.35 | 26.25 |

Table S7: Proportion of JES!-YEH! Participants with exposure levels above 95th percentiles (P95) for perfluoroalkyl substances in plasma and bisphenol A in urine for the Canadian Health Measure Survey Cycle 5 (2016-2017)

| **Contaminant** | **Age group**  **(years)** | **P95 (CI) in CHMS Cycle 5** | **Proportion of JES!-YEH! participants with exposure levels above the P95 of CHMS cycle 5**  **N (%)** |
| --- | --- | --- | --- |
| PFNA (μg /L) | 3-5 | 1.5 (1.2-1.8) | 28 (78%)* |
|  | 6-11 | 1.3 (0.76-1.8) | 49 (67%)* |
|  | 12-19 | Data too unreliable to be published | - |
| PFOS (μg /L) | 3-5 | 5.5 (3.2-7.8) | 0 |
|  | 6-11 | 4.2 (3.8-4.7) | 0 |
|  | 12-19 | 3.9 (3.7-4.2) | 1 |
| PFOA (μg /L) | 3-5 | 3.6 (2.4-4.7) | 0 |
|  | 6-11 | 2.4 (2.0-2.9) | 0 |
|  | 12-19 | 1.9 (1.4-2.4) | 0 |
| PFHxS (μg /L) | 3-5 | 3.1 (1.0-5.1) | 0 |
|  | 6-11 | Data too unreliable to be published | - |
|  | 12-19 | 3.6 (3.0-4.3) | 0 |
| BPA (μg /g creatinine) | 3-5 | Data too unreliable to be published | - |
|  | 6-11 | 5.0 (2.9-7.0) | 14 (19%)* |
|  | 12-19 | 3.2 (2.1-4.4) | 20 (26%)* |
| *Statistically significant difference in proportion of JES!-YEH! participants with values above the CHMS 95^th^ percentile (Chi-square p-value < 0.05). | | | |

References:

1. Domingo JL, Jogsten IE, Eriksson U, et al. (2012) Human dietary exposure to perfluoroalkyl substances in Catalonia, Spain. Temporal trend. *Food Chem* **135**, 1575–1582.

2. Eriksson U, Kärrman A, Rotander A, et al. (2013) Perfluoroalkyl substances (PFASs) in food and water from Faroe Islands. *Environ Sci Pollut Res Int* **20**, 7940–7948.

3. Haug LS, Salihovic S, Jogsten IE, et al. (2010) Levels in food and beverages and daily intake of perfluorinated compounds in Norway. *Chemosphere* **80**, 1137–1143.

4. Ostertag SK, Chan HM, Moisey J, et al. (2009) Historic dietary exposure to perfluorooctane sulfonate, perfluorinated carboxylates, and fluorotelomer unsaturated carboxylates from the consumption of store-bought and restaurant foods for the Canadian population. *J Agric Food Chem* **57**, 8534–8544.

5. Tittlemier SA, Pepper K & Edwards L (2006) Concentrations of Perfluorooctanesulfonamides in Canadian Total Diet Study Composite Food Samples Collected between 1992 and 2004. *J. Agric. Food Chem.* **54**, 8385–8389. American Chemical Society.

6. Vestergren R, Berger U, Glynn A, et al. (2012) Dietary exposure to perfluoroalkyl acids for the Swedish population in 1999, 2005 and 2010. *Environ Int* **49**, 120–127.

7. Wang J, Shi Y, Pan Y, et al. (2010) Perfluorinated compounds in milk, milk powder and yoghurt purchased from markets in China. *Chin. Sci. Bull.* **55**, 1020–1025.

8. Halldorsson TI, Fei C, Olsen J, et al. (2008) Dietary Predictors of Perfluorinated Chemicals: A Study from the Danish National Birth Cohort. *Environ. Sci. Technol.* **42**, 8971–8977. American Chemical Society.

9. Wu XM, Bennett DH, Calafat AM, et al. (2015) Serum concentrations of perfluorinated compounds (PFC) among selected populations of children and adults in California. *Environ Res* **136**, 264–273.

10. Park SK, Peng Q, Ding N, et al. (2019) Determinants of per- and polyfluoroalkyl substances (PFAS) in midlife women: Evidence of racial/ethnic and geographic differences in PFAS exposure. *Environ Res* **175**, 186–199.

11. Sungur Ş, Köroğlu M & Özkan A (2014) Determinatıon of bisphenol a migrating from canned food and beverages in markets. *Food Chem* **142**, 87–91.

12. Cao X-L, Perez-Locas C, Dufresne G, et al. (2011) Concentrations of bisphenol A in the composite food samples from the 2008 Canadian total diet study in Quebec City and dietary intake estimates. *Food Addit Contam Part A Chem Anal Control Expo Risk Assess* **28**, 791–798.

13. Cao X-L, Kosarac I, Popovic S, et al. (2019) LC-MS/MS analysis of bisphenol S and five other bisphenols in total diet food samples. *Food Addit Contam Part A Chem Anal Control Expo Risk Assess* **36**, 1740–1747.

14. Santonicola S, Ferrante MC, Murru N, et al. (2019) Hot topic: Bisphenol A in cow milk and dietary exposure at the farm level. *J Dairy Sci* **102**, 1007–1013.

15. Grumetto L, Gennari O, Montesano D, et al. (2013) Determination of five bisphenols in commercial milk samples by liquid chromatography coupled to fluorescence detection. *J Food Prot* **76**, 1590–1596.
